# The Artificial Moral Advisor. The “Ideal Observer” Meets Artificial Intelligence

**DOI:** 10.1007/s13347-017-0285-z

**Published:** 2017-12-08

**Authors:** Alberto Giubilini, Julian Savulescu

**Affiliations:** 1 Oxford Martin School, University of Oxford, Oxford, UK; 2 Uehiro Centre for Practical Ethics, University of Oxford, Oxford, UK

**Keywords:** Moral artificial intelligence, Ideal observer theory, Moral psychology, Moral enhancement, Artificial moral advisor

## Abstract

We describe a form of moral artificial intelligence that could be used to improve human moral decision-making. We call it the “artificial moral advisor” (AMA). The AMA would implement a *quasi*-relativistic version of the “ideal observer” famously described by Roderick Firth. We describe similarities and differences between the AMA and Firth’s ideal observer. Like Firth’s ideal observer, the AMA is disinterested, dispassionate, and consistent in its judgments. Unlike Firth’s observer, the AMA is non-absolutist, because it would take into account the human agent’s own principles and values. We argue that the AMA would respect and indeed enhance individuals’ moral autonomy, help individuals achieve wide and a narrow reflective equilibrium, make up for the limitations of human moral psychology in a way that takes conservatives’ objections to human bioenhancement seriously, and implement the positive functions of intuitions and emotions in human morality without their downsides, such as biases and prejudices.

## Introduction

Suppose you need to bin your empty cup. Because you have an ethical commitment to respecting the environment, you want the cup to be recycled. Although your moral commitment is fairly simple, you would often not be capable of making what, according to your own moral standards, would be the right choice. You might not know exactly what material the cup is made of, or whether the waste of a particular bin is destined to an efficient recycling industry. Gathering all this information and making a decision based on it requires time and cognitive resources that are frequently unavailable. Thus, you would often throw the cup in the wrong bin, falling short of your own moral standard.

You might be committed to social justice, or to alleviating animal suffering. So you might want to buy authentically fair trade products, or genuinely free range eggs. But when you are at the supermarket shelf, you do not have the time and the mental resources to gather and process all the information you would need in order to choose consistently with your principles. You might buy products based on the images or colors on the packaging, but again your choice would often fall short of your own moral standard.

Most of us probably think that it is impermissible to infringe on people’s privacy or liberty through unnecessary release of personal medical data or unnecessary quarantine measures, when different means to prevent disease outbreaks are available in principle. However, people often fail to consistently apply this basic ethical principle. Outbreaks of bubonic plague in Zaire and India in the 1990s led to military quarantines, which were not enforced in the case of HIV, tuberculosis, and malaria, despite their higher morbidity and mortality rates (Selgelid and Enemark 2008). Since people “react more strongly to infrequent large losses of life than to frequent small losses” (Slovic et al. 1980, p. 208), the effects of familiar and slow acting diseases “do not concentrate the minds of people and politicians as readily as an unfamiliar and sudden outbreak crisis” (Selgelid and Enemark 2008).

These assorted examples all display a simple truth about humans: we are often incapable of making choices consistent with our own moral goals, particularly when we have to decide in short time or in emergency situations. Thus, emotive and intuitive judgments often replace information collection, reflection, and calculation (Kahneman and Tversky 1984; Slovic et al. 2002; Haidt 2012). In short, we are suboptimal information processors, moral judges, and moral agents.

We are *suboptimal information processors* in the sense that we often fail to consider all the information required to make a rational or a moral decision. Sometimes we cannot have that information available because we do not have enough time or cognitive resources. Sometimes we have the information but we fail to give it proper consideration because of the influence of intuitions and emotions. For example, it has been shown that fearing a certain outcome makes us deem it more probable than it actually is (Peters and Slovic 1996).

We are *suboptimal moral judges* in the sense that we often fail to stick with our own consciously held moral principles. For example, even the judgments of people who are committed to egalitarian principles are often distorted by racist intuitions (Monteith 1993, Monteith et al. 2002). Often this mechanism is rooted in our neurobiology. Implicit racial bias, for instance, may be associated with noradrenaline activity in the brain (Terbeck et al. 2012).

Finally, we are *suboptimal moral agents* in the sense that, even when we can make (what we think is) the best judgment on the basis of all the relevant information, weakness of the will or particular neurophysiological states can prevent us from acting accordingly. We might in principle be opposed to violence, but our motivations are under many physiological influences (often out of our control) which make us aggressive, such as serotonin hypofunction (Seo et al. 2008) or low blood glucose (Bushman et al. 2014).

In this paper we describe a form of “moral artificial intelligence” (Savulescu and Maslen 2015), i.e., a type of software that would give us moral advice more quickly and more efficiently than our brain could ever do, on the basis of moral criteria we input. For example, it would tell us where exactly to throw our empty cup once we have told it that we want to make an environmentally friendly choice, or which eggs to buy once we have told it we care about animal welfare. More generally it would assist us in many ethical choices where—because of our cognitive limitations—we are likely to fall short of our own moral standards. Not only would this moral artificial intelligence have practical applications that would make our judgments and actions more consistent with our explicit moral goals, it would also be a philosophically interesting technology as it would implement a *quasi*-relativistic version of the “ideal observer” famously described by Roderick Firth. As a metaethical theory about the meaning of moral statements, the classical “ideal observer” theory holds that a *moral* statement (as opposed to other types of statements) is a statement to which an “ideal observer” would react in a certain way (Firth 1952, 321), for example, with a feeling of approval (Brandt 1955, 407). The ideal status of Firth’s observer is defined by its being (1) *omniscient* with respect to non-ethical facts, (2) *omnipercipient* (i.e., it is capable of visualizing, imagining, and using all the information simultaneously), (3) *disinterested*, (4) *dispassionate*, (5) *consistent*, and (6) normal in all other respects.

To the extent that we are incapable of gathering or using all the relevant information and of being impartial, immune from the distorting influence of some emotions and consistent in our judgments, we are quite far from the ideal status of Firth’s observer. Actually, one of the typical objections raised against Firth’s theory has been that the ideal observer cannot be human; therefore, his model is of no use in defining human morality. As put by Harrison, “it is quite certain that nothing answers to the description of an ideal observer which Professor Firth has given” (Harrison 1956, 256). But if it is true that there can be no human ideal observer, an artificial moral advisor (AMA) could be created to improve our moral decision-making so that we might better approximate the ideal observer, or so we shall argue.

Another typical objection made to the ideal observer theory is that it does not take in due consideration the moral role of emotions, which are not among the six aforementioned characteristics that define the ideal status of Firths’ observer (e.g., Harrison 1956, 260). Actually, according to Firth, the ideal observer would “react in a certain way” to a moral statement, which seems to suggest that emotional responses do have a role to play in his theory, at least if we assume that “reacting in a certain way” includes some feeling of approval, as for instance Brandt (1955, 407) interpreted Firth’s theory. However, it might be replied that the role of emotions in morality goes beyond merely approving or endorsing statements. According to emotivism, for example, ethical statements are themselves, as a matter of the meaning of moral terms, an expression of certain emotions, rather than statements that are merely “approved” by moral agents. While we will not take a stand on the *metaethical* dispute between emotivism and rival theories, we will take seriously the *psychological* fact that emotions play a fundamental role in the way we *make* moral judgments. However, we will argue that what determines our moral judgments is not emotion per se, but the functions performed by certain emotions. We argue that the AMA would do a better job than human emotions in fulfilling *the very same functions* that such emotions fulfill—in suboptimal ways—in human morality. And, moreover, it could advise on appropriate moral reaction.

In the following pages, we will describe how an AMA could work in practice (section 2), present the similarities and differences between the AMA and Firth’s ideal observer (section 3), explain what kind of expertise is involved in our conception of the AMA (section 4), explain in what sense the AMA can be a form of “external” moral enhancement which could be accepted by many “bioconservatives” who oppose moral bioenhancement (section 5), and finally defend our proposal from some objections (section 6).

## Towards an Artificial (Quasi-)Ideal Observer

Imagine a type of software capable of telling us, every time we have to make a moral decision, what we ought to morally do if we want to comply with certain moral principles. The software is a type of artificial intelligence capable of gathering information from the environment, processing it according to certain operational criteria we provide—for example, moral criteria such as moral values, goals, and principles—, and of advising on the morally best thing to do, i.e., on which option best meets our moral criteria (Savulescu and Maslen 2015). In this way, the software would perform all the activities that would allow us to make (nearly) optimal moral choices but that we, as humans, usually do not and/or cannot perform because of lack of the necessary mental resources or time (for instance going through all the possible options and assessing all the expected utilities of our possible choices).

It is worth noting that, in fact, we (i.e., people living in technological advanced societies) already make wide use of software to assist our decision-making and judgments. Often such technologies are based on ambient intelligence, i.e., a form of artificial intelligence that mainly works through “context awareness” (Curran 2011). In other words, it can “recognize and comprehend” the physical entities of the environmental and user’s context by modeling them through digital representations and continuous interpretation and classification of the information represented, and it can “respond” through interaction with humans in order to define and serve specific requirements (Charalampidou et al. 2012).

A rudimental version of ambient intelligence most of us use every day is a simple app for our smartphone. Suppose that I want to find a restaurant that is relatively close to my current location, relatively cheap, with reasonably good food. Many apps exist that can be used for this purpose. These apps would probably make use of some mapping technology (for example, the one used by *Google Maps*) that would provide me with a modelization of the environment (say a map of the city) based on information relevant to my needs (say a map that highlights the exact locations of the restaurants that satisfy my criteria). If we abstract from the specific example, we can describe the relevant functions of this software by saying that it:

1. *takes information* from the environment (the structure of the city),
2. *models* it according to criteria provided (the information is translated into a readable map),
3. *performs a specific task* according to the instructions (designs the map according to the locations of restaurants with certain characteristics) and
4. provides an *output* signal according to the criteria and instructions (the location of close, cheap restaurants with decent food).

IBM Watson is a cognitive technology that works through the same types of functions. Watson software is capable of interpreting human natural language and, by accessing a wide range of digitalized information, assisting humans in many areas where complicated decisions need to be made. Watson can very quickly generate and evaluate hypotheses by parsing data gathered from different sources, and can learn from its previous experience with relevantly similar types of information and tasks.^1^ Watson can be used, for instance, to assist doctors in formulating diagnosis and making clinical decisions. The software would “listen” to patients or doctors describe symptoms, cross-reference them with relevant information, such as a database of medical conditions and relevant facts about family history, and provide diagnosis or medical advice. Again, the process is the same as the one described above: the software *takes information* from the environment (patients’ descriptions), *models* information according to the criteria provided (i.e., interprets the input stimulus in a way that allows to cross-reference it with a medical database), *performs a specific task* according to the instructions provided (cross-references information about symptoms with other type of information considered relevant, for instance a medical database), and provides an *output* signal according to the criteria provided (a diagnosis and/or piece of advice).

The examples we have considered so far did not contemplate any kind of moral input. But the same kind of technology and functions could be used—so we suggest—to assist us in making decisions based on moral criteria and moral instructions.

Suppose that I do not want to find just a restaurant that is close, cheap, and with good food. Suppose that I am concerned for animal wellbeing and that I want to make ethical choices in line with this commitment. In that case, what I might want to find is a restaurant that serves (only) vegetarian food, or a restaurant that only serves meat from non-intensive animal farming. The type of operations I would ask my application to perform here is not different from the type it would have to perform in the cases described above. The difference is that this time, one of the criteria I want the software to consider, as well as my requirement regarding the type of output I want, is moral in nature, at least according to my conception of morality. The operation reflects one of my own moral values and one of the principles I would like to inform my practical decisions. Normally, there are good chances that I would not make the optimal decision. For example, it has been demonstrated that people’s choices of foods and restaurants are often determined by irrelevant factors that intuitively, but erroneously, seem consistent with their aspiration to health or ethical goals, such as the restaurants’ names, or the color of the sign, or the way dishes on the outside menu are described (Tangari et al. 2010, Chandon and Wansink 2007). It would be very difficult for me to gather and take into account enough relevant information to make what I would consider an “ethical” choice.

The Humane Eating Project in the USA has already developed an app for smartphones that does precisely what we suggested: it helps people find restaurants with—as they say—“sustainable” food options,^2^ by which they mean restaurants serving vegan, vegetarian or humanly raised meat dishes. This is an example of a kind of technology already available that, in fact, helps humans be better moral agents according to their own moral standards. And the potential for future developments is worth exploring. For example, to recall the cases introduced in the beginning, I might be strongly committed to social justice, and so I might want to find a restaurant that serves fair trade food, or guarantees adequate pay to its employees. Someone who is committed to environmental sustainability might want to know whether throwing her empty cup in that bin down the road would be an environmentally friendly choice, based on information on whether and how the trash from that bin will be collected for recycling. To the extent that such information can be made available in a way that can be modeled and used by software, an app could gather and process this type of information from the external environment, thus assisting us in making an ethically informed choice in many contexts. For instance, to recall the last example in the introduction, it could assist policy makers in making rational decisions in public health emergency situations. A computer could immediately gather information about morbidity rates of a certain disease, provide estimates of the likely spread of the disease according to information about relevant environmental factors that might affect the severity of contagion, and advise not just about the most effective policy option, but also about the most effective option that complies to the greatest extent possible with minimal ethical requirements (e.g., respect for individual privacy or autonomy in the case of quarantine).

Notice that the information gathered and processed is not limited to current states of affairs. Take for example expected utility, something for which, as discussed above, humans have often to rely on heuristics rather than on calculation. The software could calculate expected utilities of different possible outcomes in a more reliable way. The AMA of a strict utilitarian would base its moral advice just on this information, whereas the AMA of other people would provide a piece of moral advice based on the relative weight of expected utility in their moral views, compared with other ethical criteria (for example, religious ones).

There are other types of functions this software could be programmed to perform. For example, it might be programmed to ask the agent a set of questions about what criteria she wants to use on a specific occasion. In this way, the agent could clarify to himself and to the AMA her own moral goals. Many people do not have a “moral theory,” or at least not one that is consciously endorsed. With a few exceptions, people are not, or at least do not consider themselves, to be “utilitarian,” or “Kantian”. Of course some people would not even know what these words mean. It would therefore be difficult for them to instruct the AMA with precise ethical operational criteria. But certainly different people do have different moral approaches and values, even if they cannot clearly formulate them. The AMA could ask the agent a set of questions about what the agent considers morally appropriate in different circumstances, memorize and elaborate these answers, and work out a set of moral rules that are appropriate for that agent, which could be used to provide personalized moral advice in the future (for a model of how an advisor system of this kind might work in practice, see Anderson et al. 2006). For example, when I want to find a good restaurant, the AMA could be programmed to ask me if I want any particular criterion to be taken into account—animal welfare, fair trade food, etc.—offering me a range of possible ethical criteria among which to choose. On the basis of my answer, the AMA would record my moral principle and would select for me what I would consider the most ethical restaurant in my future choices. In this way, through suggesting different ethical possibilities, the AMA could facilitate education, growth and moral development.

The software might also be shared with some other people around us, such as our partner or close friends or people we particularly care about, so that others’ preferences or ethical values could be taken into account when making choices that affect them. For example, if my partner is vegetarian or committed to buying fair trade food, the AMA might take these criteria into account or remind me about them when I have to make choices that affect my partner, such as booking a restaurant or doing grocery shopping. It could, in principle, be linked to other people with similar values, learning from their choices.

Additionally, the AMA might be programmed to provide the agent not just with the best piece of moral advice, but with a range of options, signaling the one which more closely complies with the agent’s moral standards but also presenting a rank of other options based on the degree of compliance with such standards.

## The Artificial Moral Advisor and the Ideal Observer: Similarities and Differences

Firth’s ideal observer had to be *omniscient and omnipercipient* about non-ethical facts, rather than just know the morally relevant facts. Providing a criterion for moral relevance would have undermined the entire enterprise of defining “morality” by assuming a certain definition, thus begging the question of what constitutes right moral judgment. Our AMA, however, has to address a practical problem about how to provide the best moral judgment. As a consequence, a criterion for selecting the relevant information is necessary if we want the artificial ideal observer to be an operative system, rather than a theoretical construction. Our artificial moral advisor leaves the question of what counts as “moral” to the human agent, according to his or her own values. The moral agent’s moral parameters would provide the necessary criteria for moral relevance. The AMA would therefore lack one important feature that defines the “ideal” character of Firth’s observer. It would not be an “absolute” observer (Firth 1952) if by “absolute” we mean, as Firth did, an observer whose ethical statements do not contain—either explicitly or implicitly—egocentric expressions (“I like,” “I believe,” “some people think,” etc.) (Firth 1952, 319). Each moral agent would of course have different parameters with which to instruct the AMA, and this would be enough to make the statement egocentric, and therefore relativist, at least in the following sense: any moral statement of the form “you ought to do x” produced by the AMA could be expressed by another judgment of the form “if *these* are your principles [for example alleviating animal suffering] then you ought to do *x* [you ought to go to *that particular* restaurant] .” Allowing the AMA to be relativist is necessary if we want not only to render humans better moral judges, but also to respect their autonomy as moral judges and moral agents. For this reason, and because a similar relativistic connotation would “retain at least most of the other characteristics of the analysis Firth advocates—being relational, dispositional, empirical, and objectivist” (Brandt 1955, 408–9), this relativistic AMA might be preferable to the absolutist ideal observer. People have different views about what counts as “moral,” and as a consequence, different AMAs would gather different types of factual information, depending on the different criteria for the moral relevance of facts (liberal, conservative, utilitarian, deontological, mixed versions of these or others). The AMA would make use of all the information and processing capacities that we would *ideally* need in order to judge and act according to *our own* moral goals, but that our less than ideal moral psychology frequently replaces with untrustworthy intuitions and emotions.

The AMA would be *disinterested*, in the sense that its moral judgments would not prioritize any particular thing, individual or course of action merely in virtue of their particular character, *unless specifically instructed to do so*. If the instruction provided by a human moral agent aims at prioritizing some particular interests of the agent, this would not detract from the ideal character of the AMA. Simply, in that case the AMA would be performing a practical task that is not consistent with definitions of “morality” based on disinterest, or that is consistent with immoral goals, e.g., egoistic ones. Both the relativist and disinterested aspects of the AMA raise some worries about how a malevolent agent could use this technology to pursue morally bad goals. This type of objection will be addressed in section 5 below (as objection ii). It is important to notice, however, that the AMA can also play an important—although indirect—role in getting people to reflect about their fundamental moral principles and even to change them, as we explain in our response to objection i in section 5.

The AMA would also be *dispassionate*, i.e., unaffected by emotions (including particular virtues like love or compassion), exactly like Firth’s ideal observer. The AMA would only perform cognitive functions (gathering, modeling, interpreting, and processing information) that do not require the role of emotions. Independence from the distorting influence of emotions is exactly one of the aims of AMA, and one of the reasons for considering the option of an AMA, given the limits of intuitions and emotions in human moral decision-making. Independence from emotions is, however, also one of the most criticized points of the ideal observer theory in moral philosophy, because emotions are considered by many—including today, many moral psychologists and neuroscientists—to be an essential component of morality. We will address this point in the next section. The dispassionate character of the AMA does not exclude, however, that the AMA might counsel on what type of emotions to foster or at least to display in any given situation, and how to generate them. For example, it might advise that sympathy and compassion are the appropriate emotional responses when in the presence of other humans suffering (and provide psychologically engaging cues to encourage this). The AMA could council as to when anger is an appropriate response or even an appropriate motivation to take action, for example against some deep injustice we are witnessing. But even in these cases, we would still need a dispassionate point of view like the one provided by the AMA in order to have a reliable assessment of what emotions, if any, are appropriate in any given circumstance.

The AMA is also *consistent* in its judgments in the sense that, when instructed with the same moral criteria in circumstances where it would select the same factual information as relevant, and is requested to provide the same type of advice (for example about the most “ethical” restaurant in a given area), the output will be the same. This is a practical implication of the notion of “consistency” as attributed by Firth to his ideal observer (Firth 1952, 340–4).

The last requirement for the ideal observer was that it had to be *normal* in all other respects, by which Firth meant that it has to be like an average human person. The AMA would not be like a human person in any other respects, of course, because in fact it would not be a person. However, the AMA would be used by normal persons to make moral judgments and decisions, and therefore the integrated system human agent-AMA would be “normal” in all those respects that are not delegated to the AMA.

## The Expertise of the Artificial Moral Advisor

All of us, more or less consciously, often rely on the authority of “moral experts,” which are normally people we think highly of. All of us at some point have found ourselves asking what this certain person would think or do in a certain situation. The software we are proposing would play the role of such a moral expert, but it would be an expert more informed and more capable of information processing than any other human moral expert we trust. Consider the following example provided by Peter Singer to support his point that certain people, namely professional ethicists, can be considered “moral experts”:

“I may […] be wondering whether it is right to eat meat. I would have a better chance of reaching the right decision, or at least, a soundly based decision, if I knew a number of facts about the capacities of animals for suffering, and about the methods of rearing and slaughtering animals now being used. I might also want to know about the effects of a vegetarian diet on human health, and, considering the world food shortage, whether more or less food would be produced by giving up meat production. Once I have got evidence on these questions, I must assess it and bring it together with whatever moral views I hold. Depending on what method of moral reasoning I use, this may involve a calculation of which course of action produces greater happiness and less suffering; or it may mean an attempt to place myself in the positions of those affected by my decision; or it may lead me to attempt to "weigh up" conflicting duties and interests” (Singer 1972, 116).

The kind of moral expert Singer had in mind was a human being, but, considering the type of mental processes he describes, a type of software like the AMA could perform the same functions in a more efficient and reliable way.

We could imagine a pool of moral experts being consulted when programming the AMA. The moral experts could decide which basic moral principles, or constraints, should be put in the AMA as basic filters. Within these limits, different moral agents would then be able to use the AMA to pursue their different moral goals, thus promoting a pluralism of ethical views within reasonable moral boundaries.

But we could also imagine moral experts inputting more specific criteria in the AMA, in the form of more substantial moral principles specific of a certain moral approach (always within the boundaries of the basic moral constraints). For example, we could imagine a Catholic moral expert inputting criteria that would help an agent complying with the Catholic doctrine, or an expert utilitarian philosopher helping programming the AMA with utilitarian principles. Every moral agent might be able to choose which version of the AMA to use, without having to decide by her/himself which specific principle to use to instruct the AMA. For example, some people just want to be good Catholics, but do not know exactly what principles to apply in different circumstances. A Catholic AMA might assist them in behaving consistently with their basic moral commitment, and the same applies to other moral approaches.

## Artificial Moral Enhancement as an Alternative to Bioenhancement: the Challenge to Bioconservatives

Some philosophers have suggested that it is necessary to intervene through biomedical means—namely genetic selection, genetic engineering, and/or pharmaceutical drugs—to enhance moral decision-making, either through “moral enhancement” (Persson and Savulescu 2011; Kahane and Savulescu 2013) or through “cognitive enhancement” (Harris 2010). According to the former, we need to enhance our moral dispositions, i.e., modulate certain neurophysiological states, including emotions, for example, through administration of substances like serotonin to modulate aggressiveness or propranolol to reduce racial bias. According to the latter, we need to increase our cognitive capacities so as to let reasons, instead of emotions, guide our judgments and behaviors. These (as well as other) forms of human bioenhancement have been opposed on the basis of some typically “conservative” objections (Giubilini and Sanyal 2015). It has been claimed that human bioenhancement does not leave us “open to the unbidden” and thus does not allow us to fully appreciate the gifted character of our life (Sandel 2004), that it compromises our “authenticity” (Elliott 1999) or human dignity (Fukuyama 2003, Cohen 2006), that it fails to preserve what is of value in human nature, including its natural limitations (Cohen 2006). Interestingly, their target is mainly not the intended outcomes of the proposed enhancement interventions, but the intrinsic wrongness of the biomedical means proposed to achieve such outcomes. No moral conservative is opposed to making people more intelligent or more moral through “traditional” means, e.g., by giving everybody access to a good education system or learning tools (such as computers). Our proposed AMA would represent a challenge for bioconservatives. The AMA would be an external technology—or “artificial moral enhancement”—which humans can decide whether to use or not, exactly as we do with computers, with all the benefits of a human enhancement without the drawbacks (assuming for the sake of argument that there actually are drawbacks) which worry bioconservatives.

This is not to say that the AMA would be “preferable” to either moral or cognitive bioenhancement. Whether or not it is depends on whether those conservative objections are valid or not, an issue which is beyond the scope of this paper, and on the precise risks and benefits of each intervention. But the AMA would shift the burden of proof back onto bioconservatives: they would have to show why this particular form of technological moral enhancement would be impermissible. Until then, we can say that another advantage of our proposal is that it takes some of the conservatives’ objections to human bioenhancement seriously, because our artificial moral enhancement does not involve “playing god” with human nature.

Another objection that moral *bio*enhancement might face is the “bootstrapping problem” (Weisberg 2012): if we enhance our moral capacities through interventions on our neurophysiology, we would have no independent standpoint from which to assess the success of the enhancement. The capacity to constantly monitor, assess, and have control over modification of moral dispositions is very important, if we consider that changing certain moral dispositions could change our moral psychology in undesirable ways, for example, making us *too* utilitarian, or *too* empathetic (Agar 2013). Because our ability at introspection is limited, we might not be able to rationally and objectively assess whether a certain moral disposition has been enhanced in the desired (and desirable) way. An objective evaluation of a certain type of moral bioenhancement would require an independent standpoint from where to assess the moral disposition. Note however that this is not an objection to moral bioenhancement per se, as the same objection would apply to any unenhanced individual who presumably uses her/his own moral standard to improve her/his own dispositions in more traditional ways (e.g., through education). The AMA would in any case offer a better option for those who think that self-assessment is a problem for both the enhanced and the unenhanced. Insofar as judging something external to us gives us an advantage over judging our own dispositions, the AMA would have the advantage of not requiring introspection to be assessed, since there is separation between the assessor and the enhanced mechanism. Admittedly, this is not enough to resolve the bootstrapping problem: the AMA would be instructed with our own moral parameters, so the moral criteria with which we would assess its responses are the same as those it has used to provide such responses. However, it is foreseeable that at least on some occasions the AMA would return responses that are counterintuitive and that we might not be disposed to accept. Indeed, it could prompt responses which other moral advisor systems have made. In such cases, the AMA would force us to reflect upon our own moral criteria and our own intuitions, if not to change them. In this way, it could favor self-assessment in a way that neither moral bioenhancement nor traditional forms of enhancement, e.g., education, allow. We examine more closely this aspect in addressing the first of a series of possible objections to the AMA in the next section.

## Objections

We proceed now to examine and address some likely objections against our proposal. Some of these objections can also be raised against Firth’s theory, while others only apply to the AMA. We will either argue that the objection in question is not valid or not strong enough; or we will take the point of the objection and use it to refine our proposal by suggesting how the AMA could be modified to address the objection.

### Objection (i): the AMA Would Return Counterintuitive or Overdemanding Responses

Presumably, the AMA would at times return responses that are counterintuitive. This is only to be expected, if the AMA is meant to make up for the limitations of our intuitive and emotive moral psychology. One might think that the AMA would be of no use in such cases, because agents would not be willing to endorse or act upon a counterintuitive moral judgment. However, in this way the AMA might prompt us to balance our intuitions against the piece of advice, and vice versa, to attain a condition of “reflective equilibrium,” which can be understood in the narrow or in the wide sense. In the narrow sense, reflective equilibrium requires a certain degree of coherence between one’s considered judgments and principles within a certain moral perspective (for example, an egalitarian, or utilitarian, or religious perspective). In its wide sense, reflective equilibrium requires balancing different moral perspectives against one another (e.g., a utilitarian against an egalitarian or a deontological or a religious) (Daniels 2013, Mikhail 2010, Rawls 2001). However, most of us are incapable of balancing different competing fundamental principles; for example, recent findings in moral psychology suggest that liberals and conservatives base their moral approaches on different sets of intuitive and emotive foundations almost inaccessible to reason (Haidt 2012, Haidt and Graham 2007).

Here is where an apparent objection to AMA—i.e., the possibility of counterintuitive responses—turns out to be one of its strengths. The AMA’s response would be an external contribution towards reaching a condition of reflective equilibrium. More precisely, the AMA could help reach a *narrow* reflective equilibrium because the counterintuitive response would introduce a new element in the dynamics through which we balance our considered judgments and our principles. A counterintuitive response from the AMA would prompt us to question our considered judgments or some of our principles, adjust either or both according to the counterintuitive responses, and/or adjust the counterintuitive response from the AMA, for example by taking an attenuated version of it.

Here is an example. If I want to be altruistic in a utilitarian way, i.e., in a way that is maximally effective and impartial (Singer 2011), I would be often advised by the AMA to do things that my intuitions would deem supererogatory, like giving most of my income to charities. In such cases, I might want to slightly adjust my moral principles and/or considered judgments in a more altruistic direction, e.g., convince myself that giving a substantial part of my income to charities is a moral duty, and/or take a milder version of AMA’s advice and agree to give, say, just a third, rather than a half, of my income to charities.

The contrast between the ideal observer’s advice and my intuitive judgment could also help us achieve a *wide* reflective equilibrium. The counterintuitive responses can lead to question our fundamental moral views and balance them not only against the counterintuitive responses, but also against alternative moral views. For example, the counterintuitive advice provided by the AMA instructed with utilitarian operational criteria might convince me that after all, utilitarianism is not a moral theory I want to subscribe to because of its overdemandingness. Operational criteria consistent with different moral theories or approaches could be memorized by the AMA and used for future decisions, so that the pieces of advice I would eventually receive would be closer to the balance of different moral views that are consistent with my considered judgments.

Thus, a counterintuitive response resulting from utilitarian operational criteria would lead me to adjust my general moral views towards a milder version consistent with less counterintuitive particular judgments, for example, with a an “easy rescue” utilitarianism that would require maximizing utility as long as this is not achieved at too high a cost for me (Savulescu 2007), or to adopt a different moral perspective or a mix of elements from different moral perspectives encompassing, say, deontological or religious elements alongside utilitarian ones.

In addition, the AMA could prompt certain kind of reflection or empathic engagement, through using personal stories, media, games, etc.

### Objection (ii): Dangers of Relativism

Because we have hypothesized that the agent provides the operational criteria, the AMA would work equally well regardless of whether these criteria are ethical or unethical, egoistic or altruistic, of a saint or a psychopath. The contribution the AMA would give us towards adjusting our principles and judgments—discussed in the previous section—would not make us more moral unless we already have an at least basic commitment to be moral, which many people do not have.

This objection should be taken into account when programming the AMA. We propose that the AMA, while accepting the widest range of moral criteria possible, should be programmed with some basic “moral filters” that constrain the range of possible operational criteria to be used as input. We need not take a stand on the issue whether moral relativism is true or false to justify adding such constraints. For one, relativism is not the same as the view that any possible moral principle is as valid, good, true as any other (at the metaethical level), or that any moral view should be equally respected (at the normative level). Although relativism can be defined as the theory that the truth of moral judgments and principles is relative to a certain culture or group, it is not incompatible with the view that some systems of morality—understood as agreements among members of a community—are better than others (Harman 1975, 4; Wong 2006). Besides, and more importantly at the practical level, we need to put constraints on people’s behavior regardless of whether we think there are objective standards of ethics, because there are pragmatic or political ends—most notably regulating the cohabitation of different people—whose justification need not be based on metaethical theories. Western society is already organized around some basic principles—such as reciprocal respect, tolerance, protection of persons’ lives—which are enforced regardless of whether some people or moral systems acknowledge them. The AMA would simply follow the same approach we already adopt in shaping the institutions of liberal societies. We could make sure that some basic moral requirements are met while at the same time allowing people to use the AMA according to their different moral criteria. For example, the AMA could be programmed to avoid advising about killing people or stealing**.** As we suggested above, a pool of moral experts could be consulted when programming the AMA to make sure that its advice is always consistent with such basic moral principles.

To be sure, there would be cases in which killing and stealing are morally acceptable or even, one might think, morally obligatory (for instance to defend others, to save someone from starvation). These circumstances however would be very rare, and the fact that the AMA would not be reliable in such cases does not detract from its usefulness in the majority of our everyday ethical decisions that do not involve such exceptional actions as killing or stealing for a greater good. As long as human agents are informed that the AMA cannot advise about how and when to kill, and that they can autonomously decide whether or not to take the AMA’s advice, the situation in such circumstances would not be worse than it would be if the AMA were not used. Also, as technology evolves and software becomes more sophisticated, we might have an AMA capable of distinguishing those cases in which the basic moral constraints can be permissibly waived—for example, killing someone who is about to blow up a building in order to save many lives—and those in which it cannot—for example, killing in a terroristic attack.

While we believe ethical relativism is not a defensible position, beginning with a relativistic AMA would be a practical first step towards non-relativism. It would engage the individual’s own ethical code but develop that by showing the full consequences and circumstances of action, while also suggesting alternative courses of actions or moral values. In more full blown versions, it could be programmed to suggest what moral paragons or leaders had chosen in similar situations, striving towards at least a rational intersubjectivity. The AMA could even facilitate development towards more objectivist moral codes by engaging with an individual’s own morality and psychology. Ideally, it would function as a moral adviser and perhaps even persuader.

### Objection (iii): The AMA Does Not Take Emotions into Due Consideration

As a matter of fact, intuitions and emotions drive most of our moral and practical decision-making. “Emotionism,” i.e., the idea that emotions are in some way essential to morality (Prinz 2007), is not only a philosophical view (in the form of an emotivist metaethical theory), but also a psychological and neuroscientific theory which has received a lot of empirical support in recent years. Humans base most of their practical judgments on “affect heuristics,” i.e., intuitive and emotive, rapid responses (Finucane et al. 2000; Gilovich and Griffin 2002). Objects, events, and possible outcomes are often tagged with different, readily available emotions (positive or negative), which serve as cues for many important judgments we make and for which mental resources or time availability are likely to be limited. According to recent theories in moral psychology, our sense of morality is based on a limited set of intuitive and emotive responses, where principles and reasons are, more often than not, mere post-hoc rationalizations (Cushman et al. 2003, Haidt and Graham 2007, Haidt 2012). In general terms, humans “are effort-minimizing information processors inclined to adopt the simplest and least effortful processing strategy” (Forgas 1995, 46).

Consider disgust, for instance. Disgust was originally a physiological mechanism that served as a defense from poisons and parasites. It has been suggested that disgust has eventually been “co-opted” by our moral psychology (Kelly 2011) and upgraded into a more complex evaluation system which signals perceived social and moral violations pertaining to the dimension of “purity” and “sacredness” (Rozin et al. 2009), such as incest, but probably also fairness violations (Sanfey et al. 2003, Skarlicki et al. 2013). Disgust is often a useful heuristic for quickly recognizing potentially morally problematic situations that would otherwise require a lot of reflection, observation, and mental efforts for which we might not have the mental or even conceptual resources (Hauskeller 2006, 599; Kass 1997), or more specifically behaviors that, like microbes and pathogens, can be contaminating and lead to “social contagion” (e.g., heavily drinking in public) (Plakias 2013).

Intuitive and emotive responses can also result from the automatization of rational reflection or of the application of consciously held moral goals (Fishbach et al. 2003; Bargh and Chartrand 1999). Our moral principles, in other words, “may be overlearned to the point of their a) subliminal activation, and b) relative independence of cognitive resources” (Fishbach et al. 2003, 298). Also in this case, at the basis of our emotive and intuitive responsiveness is the typically human necessity to minimize cognitive resources and time.

The other side of the coin is that—as we have seen—often intuitions and emotions are also sources of biases and other types of irrational or immoral judgments that, as discussed at the beginning, make us bad information processors, bad moral judges, and bad moral agents. Some people are disgusted by the idea of homosexual sex, and indeed disgust sensitivity has been shown to be a predictor of condemnation of homosexuality (Inbar et al. 2009). But the disgust reaction is not necessarily linked to any morally relevant aspect of the practice or object being judged (Kelly and Morar 2014; Giubilini 2015)—unless, of course, we want to say that disgust is itself a reason for considering the object of disgust immoral (Kass 1997).

Thus, the very same characteristics that make emotions and intuitions an essential component of our practical and moral judgments and decisions (automaticity, independence from cognitive resources, minimization of mental effort) also make emotions and intuitions unreliable because sources of biases.

The AMA would maintain the positive functions of emotions, while avoiding the downsides. More in particular, emotions fulfill two main functions in human moral psychology. First, they are necessary but imperfect proxies for complicated reasoning and calculation for which we do not have the mental resources and for which we need heuristics. The AMA would have the advantage of providing the “real thing”—information processing, calculation, weighing of expected utilities, and so on—rather than the imperfect proxy. Second, human emotions and intuitions have the function of automatically drawing our attention to morally relevant aspects of certain situations (for instance disgust might draw our attention to anti-social behaviors like heavily drinking in public or to sexual taboos) prior to and independent of any conscious reflection. Again, as seen above, the way emotions perform this activity is inevitably suboptimal. The AMA, on the other hand, would perform the same function by immediately pointing at morally relevant aspects (according to the agent’s own standards of morality) of a certain situation that has been modeled and categorized in its software. Admittedly, some of the situations and the choices humans have to face are very complex, and in many such cases, humans’ skills are still better than any software we currently have. Our claim is not that computers are better than humans in any possible circumstance, but that work needs to (and can) be done to increase the reliability of computers as moral advisors and make them of assistance to humans in an increasingly wider set of circumstances.

Emotions are often considered to have at least two other important functions in human morality. First, in a Humean perspective, the belief that something is morally right or wrong boils down to approving or disapproving of it, where approval and disapproval are emotional attitudes. In this view, the role of emotions would be required in order for us to be able to endorse the AMA’s moral or practical advice. Admittedly, this is an aspect of an emotion based moral system that the AMA cannot replace.

Secondly, emotions are necessary to the motivational aspect of morality. They provide the indispensable link between moral judgment and moral action. It is noteworthy, for example, that without the correct functioning of certain emotions (sympathy, shame, guilt, grief) following brain damage, people with adequate knowledge of appropriate social behavior turn into psychopaths that simply do not behave as they know they should (Damasio 1994).

The distorting influence of our emotional states might be present at these two levels, making us incapable not only of accepting what would otherwise seem a reasonable moral judgment, but also of acting upon it. We propose that the AMA could be integrated with emotion and neurophysiological detection technologies in a way that could assist our moral judgments and actions by making us aware of emotional and potentially distorting factors. Consider, for example, a system for “ambient-assisted emotional regulation” (Garzo et al. 2010). The system is intended for use in elder care, and it “aims at exploring the combination of physiological emotion detection and ambient intelligence in the context of emotional regulation” (Garzo et al. 2010; see also Nasoz et al. 2003, Leon et al. 2007). The system uses software incorporated in people’s clothes that can monitor neurophysiological states; when an abnormal and potentially threatening state is detected, the system sends out a signal that prompts assistance for that person. For example, when the system detects a neurophysiological state of fear, it automatically switches on the light, or contacts emergency services. We propose the same technology could be used to notify us of an emotional state that might negatively affect our moral or practical decisions. Google is currently developing contact lenses with a microchip in them that monitors levels of glucose in the blood of diabetics (Liu 2013).^3^ Although the purpose of this project is to assist medical professionals and patients, the same technology could be used to improve human decision-making in everyday life. A recent study has shown that low glucose levels are correlated with higher aggressiveness in couples (Bushman et al. 2014). Knowing when our neurophysiological state is likely to lead to aggressive behavior may assist us in making better decisions, for example by suggesting to postpone a certain discussion or to put more effort into self-control.

A further technological help in this direction could come—so we suggest—from so-called “neurofeedback training” (NFT). NFT is the use of monitoring devices “to provide the individual with explicit information regarding specific aspects of his cortical activity in an easy-to-understand format and in doing so encourage him to alter particular target components, such as amplitude and frequency” (Vernon et al. 2009, 2). In other words, subjects can be trained to alter their brain activity in certain respects by being made aware of some neurophysiological parameters. For example, NFT has been used to reduce anxiety and improve mood (Vernon et al. 2009). But once again, we want to suggest that this technology could be used to improve our moral capacities. A recent study has shown that receiving neurofeedback about their brain activity enabled subjects to change brain network function of areas related to empathy (Moll et al. 2014). Research on neurofeedback is however still in its infancy, and its methodology and results are still debated (Vernon et al. 2009 provide a useful review of the results). However, should this methodology prove itself to be efficient, we suggest it could be used as integration to the AMA and the neurophysiology detection technologies for moral enhancement purposes.

## Conclusions

“There is no moral compass app”—so reads a slogan of a recent advertising campaign for an American university. Even in our hypertechnological world—so the slogan suggested—we cannot rely on computers to find moral answers. In this article, we have challenged this assumption by proposing a form of artificial intelligence that could assist us in making better, including better informed, moral decisions. Such moral artificial intelligence would be desirable for a number of reasons that have to do with our limited moral capacities. Evidence from moral psychology and the psychology of decision-making has suggested that intuitions and emotions drive our judgments and decisions most of the times, and often not in the desirable way (Kahneman and Tversky 1984; Haidt 2001, Gilovich and Griffin 2002, Cushman et al. 2003, Haidt and Graham 2007, Haidt and Joseph 2007, Kahneman 2011). To be sure, intuitions and emotions can be efficient heuristics in many circumstances (Finucane et al. 2000, Roeser 2010), but their overall reliability is undermined by the fact that we cannot know when they are trustworthy. We often make judgments and behave in “immoral,” or at least less than moral, ways, according to *our own* consciously held moral goals and principles.

We have called our proposed moral artificial intelligent “artificial moral advisor” (AMA) and we have shown that it can be seen as a version of the “ideal observer” described by Roderick Firth in his theory about the meaning of moral statements. The AMA would gather morally relevant information, process it and, return moral advice based on an agent’s own moral goals. We have argued that the AMA could be used to enhance our moral and practical decision-making in a way that holds promises for being more efficient and less controversial than moral bioenhancement. Our AMA differs from Firth’s ideal observer in some important respects. Most notably, while Firth’s ideal observer was an absolute observer, the AMA would be a partly *relativist* observer: its moral advice would reflect the ethical values of the particular human agent using it. This difference makes our AMA “ideal” in a sense that is slightly different from the sense in which Firth’s absolutist observer was “ideal.” However, we have argued that the difference makes our AMA preferable to Firth’s model.

Our proposal challenges (bio)conservatives to provide reasons why their typical objections to human enhancement would apply to this form of enhancement. On our part, we have provided reasons in favor of AMA, which we can synthesize as follows:

1. the AMA would meet the requirements set by the ideal observer theory in moral philosophy, and therefore it is not only a technology with practical relevance, but also a philosophically interesting experiment in that it would translate a theoretical model in moral philosophy into a real moral advisor;
2. the AMA would respect and indeed enhance individuals’ moral autonomy, as it would allow individuals to implement their own moral perspective in the best possible way, within certain basic moral constraints that AMA would be instructed to consider;
3. the AMA would help individuals achieve wide and a narrow reflective equilibrium,
4. the AMA would make up for the limitations of human moral psychology in a way that takes conservatives’ objections to human bioenhancement seriously, and
5. finally, far from being a bad surrogate of morality because of its lack of reliance on intuitions and emotions, the artificial ideal observer would represent human morality at its best because it would implement the positive functions of intuitions and emotions without the downsides. Such an advisor would not eschew basic human emotions but seek to regulate them and activate the right emotion for a given context.

To be sure, human beings have incredibly rich moral lives and are capable of noble deeds. An artificial moral advisor is not going to usurp or surpass human moral agency any time soon, though it may do so one day. For the foreseeable future, we ought to acknowledge our own moral limitations as well as our strengths, and utilize our awesome cognitive powers to develop technology to “limit our limitations” and to realize our potential as moral agents.

## Footnotes

1 The presentation of IBM Watson can be found at http://www.ibm.com/smarterplanet/us/en/ibmwatson/ (last accessed 8 July 2014)

2 See information on the Humane Eating app at America for Animals webpage http://www.Americaforanimals.org/humane—eating—project/ (last accessed 9th July 2014)

3 A presentation of this type of Google lenses can be found at http://www.iflscience.com/technology/google—seeks—integrate—cameras—contact—lenses
